# Advances on the Amaryllidacea Alkaloids Collected in South Africa, Andean South America and the Mediterranean Basin

**DOI:** 10.3390/molecules28104055

**Published:** 2023-05-12

**Authors:** Antonio Evidente

**Affiliations:** 1 Department of Chemical Sciences, University of Naples Federico II, Complesso Universitario Monte S. Angelo, Via Cintia 4, 80126 Naples, Italy; evidente@unina.iy; 2 Institute of Sciences of Food Production, National Research Council, Via Amendola 122/O, 70185 Bari, Italy

**Keywords:** Amaryllidacea, secondary metabolites, alkaloids, biological activity, Andean South America, South Africa, Mediterranean regions, chemistry

## Abstract

The alkaloids are one of the most represented family of natural occurring biological active compounds. Amaryllidaceae are also very well known for their beautiful flower and are thus used as ornamental plants in historic and public gardens. The Amaryllidacea alkaloids constitute an important group that is subdivided into different subfamilies with different carbon skeletons. They are well known from ancient times for their long application in folk medicine, and in particular, *Narcissus poeticus* L. was known to Hippocrates of Cos (ca. B.C. 460–370), who treated uterine tumors with a formulate prepared from narcissus oil. To date, more than 600 alkaloids of 15 chemical groups exhibiting various biological activities have been isolated from the Amaryllidaceae plants. This plant genus is diffused in regions of Southern Africa, Andean South America and the Mediterranean basin. Thus, this review describes the chemical and biological activity of the alkaloids collected in these regions in the last two decades as weel those of isocarbostyls isolated from Amaryllidaceae in the same regions and same period.

## 1. Introduction

**Figure 1:**
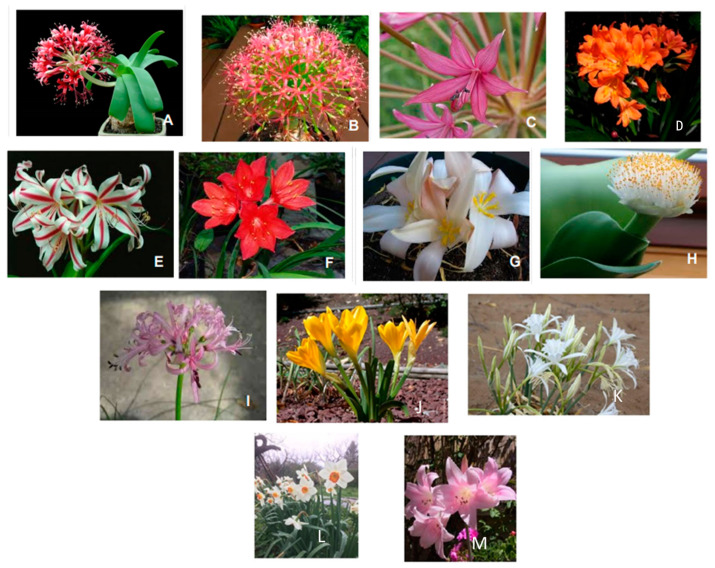
Some representative Amaryllidaceae genera [2]: (**A**) *Ammocharis coranica*; (**B**) *Boophone disticha*; (**C**) *Brunsvigia radulosa*; (**D**) *Clivia miniata*; (**E**) *Crinum delagoense*; (**F**) *Cyrtanthus contractus*; (**G**) *Gethyllis ciliaris*; (**H**) *Haemanthus albiflos*; (**I**) *Nerine filifolia*; (**J**) *Sternbergia lutea*; (**K**) *Pancratium maritimum*; (**L**) *Narcissus tazetta*; (**M**) *Amaryllis belladonna*. The pictures are original photos obtained by the author.

Amaryllidaceae plants grow as wild species in several countries and are also cultivated for their beautiful flowers and for the production of volatile oils (Figure 1). They are classified into 60 genera [1] that are diffused in different world regions but that are dominant within three distinct geographical locations, i.e., Andean South America, Southern Africa and the Mediterranean basin [2,3]. About one third of one thousand Amaryllidaceae species grow in South Africa and are commonly used in folk medicine [2]. They include well-known ornamental varieties such as daffodils (*Narcissus*), snowdrops (*Galanthus*) and snowflakes (*Leucojum*) [3]. Consequently, they have a high commercial value and are important for the floriculture industry [4,5]. The use of *Narcissus* in the Mediterranean basin begins at the time of Hippocrates and Pliny [6], while in South America in the archeological Inca ruins, there were found floral depictions of *Ismene*, *Pyolirion* and *Stenomesson* [7]. Paintings of *Brunsvigia* species were found in Lesotho [8].

**Figure 2:**
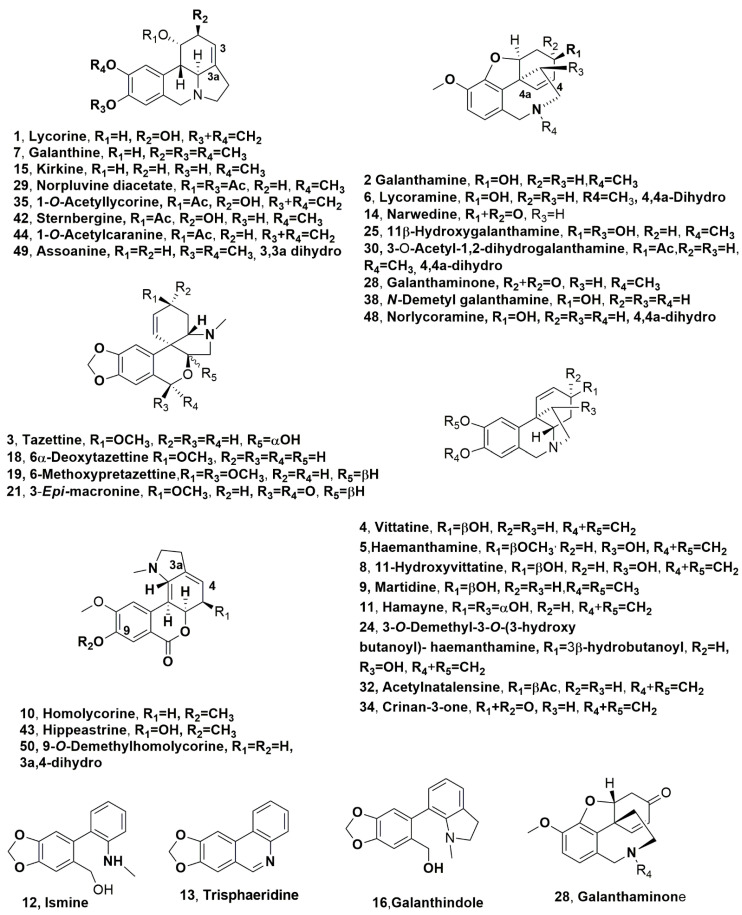
Alkaloids produced by Andean Amaryllidaceae plants: *Habranthus jamesonii*, *Phycella herbertiana*, *Rhodophiala mendocina* and *Zephyranthes filifolia* (**1**–**6**) *H. jamesonii* and *P. herbertiana* (**7** and **8**), *Caliphruria subedentata* (**1**, **2**, **5**, and **10**–**16** and **18**, **19** and **21**), *Hippeastrum papilio* (**2**, **5**, **14**, **24** and **25**) and *Rhodolirium andicola* (**2**, **5**, **6**, **18**, **21**, **28**–**30** and **32**, **34**) *Crinum amabile*, *Crinum erubescens*, *Crinum moorei*, *Amaryllis belladonna* and *Zephyranthes carinata* (**1**, and **35**), *Pyrolirion albicans* (**2**, **4**, and **38**, **42** and **43**), *Amaryllis belladonna* (**44**), and *Ismene amancaes* (**1**, **2**, **6**, **7**, and **42** and **48**–**50**).

Studies on Amaryllidaceae alkaloids (AA) began in 1877 with lycorine (**1**, Figure 2), which is the main Amaryllidaceae alkaloid that was isolated from *Narcissus pseudonarcissus* [9]. From that time, investigations of this group of alkaloids increased considering their wide range of biological activities. These include antitumor, antiviral, antibacterial, antifungal, antimalarial, analgesic, and cytotoxic activities [10]. The most important application in medicine of Amaryllidaceae alkaloids is represented by the use of galanthamine (**2**, Figure 2) to treat Alzheimer’s disease and is already commercialized as a drug. Galanthamine (**2**) is able to selectively inhibit the enzyme acetylcholinesterase (AChE), which plays a fundamental role in the disease [1,11]. Amaryllidaceae plants also synthesize poisons such as lycorine and galanthamine, and this toxicity should be always considered [12].

Hundreds of scientific articles have reported on the biosynthesis, source, isolation and biological activities of AA, which are grouped into more than 12 subfamilies considering their carbon skeleton, including norbelladine, rystilline, α-crinanes, β-crinanes, lycorane, galanthamine, pretazettine, homolycorine, montanine, cherylline, crinasiadine, clivimine, ismine analogues and miscellaneous alkaloids [1,6].

Considering the number of scientific articles describing the different aspects of AA, several articles were published on this topic regarding the phytochemical, biological and pharmacological properties of *Crinum bulbispermum* [13] *Nerine* [14], *Haemantheae* [15] and *Crinum*, *Ammocharis*, *Amaryllis* and *Cyrtanthus* [16]. The relation between the biological activity of AA and their absolute configuration was also extensively described [17] as well as the acetylcholinesterase inhibition of extracts of Amaryllidaceae collected in South Africa with potential for Alzheimer’s disease treatment [18]. Recently, only AA isolated in the decade 2009–2020 were reported [19]. 

This review reports on the plant sources, isolation, and chemical and biological characterization of alkaloids produced by Amaryllideceae that are native to Andean South America, South Africa and the Mediterranean basin, that were collected in the last two decades (2009–2023), and that are of the findings that resulted from SCI-FINDER. The results are chronologically reported in three different sections, namely Section 2, Section 3 and Section 4. Similarly, Section 5 reports the chemical and biological properties of isocarbostyls isolated from Amaryllidaceae in the same regions and same period.

## 2. Alkaloids Isolated from Amaryllidacae Plants Collected in Andean South America

In Argentina, about 61 species of the Amaryllidaceae family grow, covering a wide variety of genera (*Chlidanthus*, *Crinum*, *Habranthus*, *Haylockia*, *Hieronymiella*, *Hippeastrum*, *Phycella*, *Rhodophiala*, *Stenomesson* and *Zephyranthes*) [20]. In particular, almost all the wild *Habranthus jamesonii*, *Phycella herbertiana*, *Rhodophiala mendocina* and *Zephyranthes filifolia* species collected in the Argentinian Andean region contain lycorine and galanthamine (**1** and **2**). The organic extracts of all four species showed strong AChE inhibitory activity with IC_50_ values between 1.2 and 2 µg/mL. The main alkaloids found in these species were lycorine, tazettine, vittatine, haemanthamine and lycoramine (**1**, and **3**–**6**, Figure 2), which are not produced by *Z. filifolia*, while galanthine and 11-hydroxyvittatine (**7** and **8**, Figure 2) are only produced by *H. jamesonii* and *P. herbertiana* [21]. These alkaloids show other interesting biological properties besides their AChE inhibitory activity. Haemanthamine (**5**) strongly induces apoptosis [22] and exhibits antimalarial activity [23]. Vittatine (**4**) shows cytotoxicity and antibacterial activity [23], as does 11-hydroxyvittatine (**8**) [6]. Lycorine (**1**) exhibits cytotoxic, apoptotic, antiviral, antifungal, antiprotozoan [6,22], and anti-inflammatory activities [24]. Furthermore, alkaloid **2** could be a promising therapeutic agent against leukemia [25]. Galanthine (**7**) possesses analgesic and hypotensive effects [23], while tazettine (**3**) shows moderate cytotoxic activity [26].

**Figure 3:**
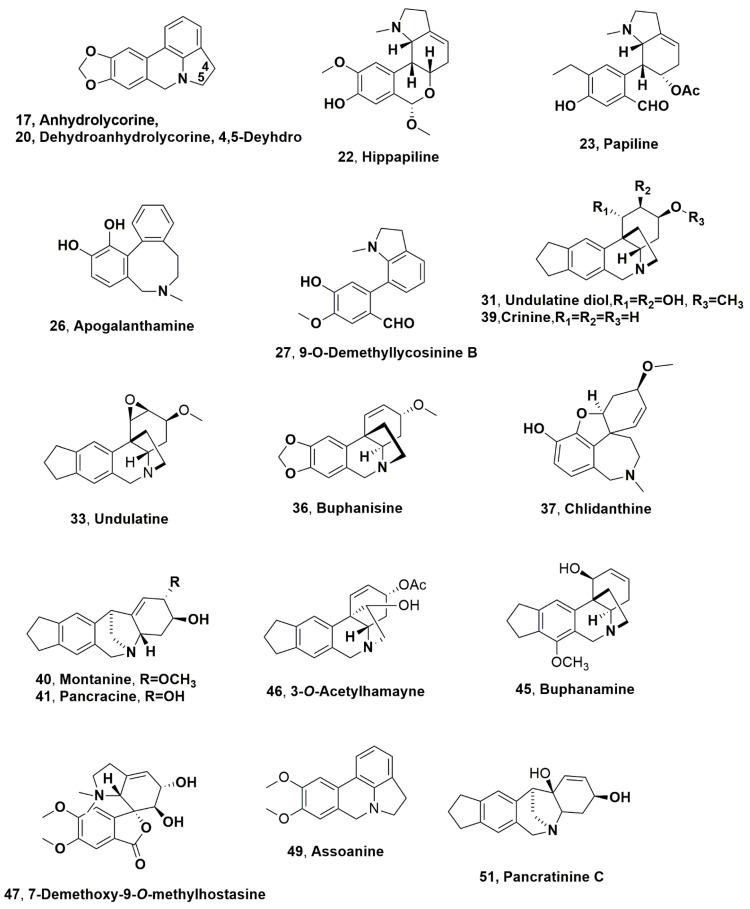
Alkaloids produced by *Caliphruria subedentata* (**17**, **20**), *Hippeastrum papilio* (**22**, **23**, **26** and **27**) and *Rhodolirium andicola* (**31** and **33**) *Crinum amabile*, *Crinum erubescens*, *Crinum moorei*, *Amaryllis belladonna* and *Zephyranthes carinata* (**20**, **33**, and **36**), *Pyrolirion albicans* (**37** and **39**), *Amaryllis belladonna* (**45**–**47**), *Hippeastrum stapfianum* (**47**), and *Ismene amancaes* (**17**, **20** and **51**).

Lycorine, galanthamine, haemantamine (**1**, **2**, and **5**), maritidine, homolycorine and hamayne (**9**–**11**, Figure 2) were isolated from the ethanolic extract of *Caliphruria subedentata*, which showed moderate cytotoxic activity [27,28]. This Amaryllidaceae as well as *Caliphruria hartwegiana* and *Caliphruria tenera* are considered endemic in Colombia [29]. They belong to the genus *Caliphruria* that is not much diffused in the world, as only four species were found in some tropical regions of South America [29], but particularly, they are localized in restricted parts of the Central and Occidental Cordilleras in the departments of Valle del Cauca, Cundinamarca and Huila [30]. The successive investigation allowed for the identification of tazzetine (**3**, Figure 2), ismine, trisphaeridine, narwedine, kirkine, galanthindole, 6α-deoxytazettine, 6-methoxypretazettine, and 3-*epi*-macronine, and anydrolycorine and dehyroanhydrolycorine, (**12**–**16** and **18**, **19** and **21**, Figure 2, and **17** and **20**, Figure 3) in the organic extract of *C. subedentata* using the GC-MS technique [31].

Hippapiline, papiline, and 3-*O*-demethyl-3-*O*-(3-hydroxybutanoyl)- haemanthamine (**22** and **23**, Figure 3, and **24**, Figure 2) were isolated together with haemanthamine (**5**), galanthamine (**2**), narwedine (**14**), 11β-hydroxygalanthamine (**25**, Figure 2) apogalanthamine, and 9-*O*-demethyllycosinine B (**26** and **27**, Figure 3) from an indigenous Brazilian species of *Hippeastrum papilio*. This Amaryllidaceae was collected during the flowering period in the south of Brazil (Caxias do Sul—RS) [32]. 

Galanthamine (**2**) was isolated from the bulb extract of *Rhodolirium andicola*, which is a native Chilean Amaryllidaceae species that was collected during the flowering season in December 2016 from National Park Conguillio, Araucanía Region, Chile [33]. This organic extract, which contained 2.3 ± 0.18 g/mL of alkaloid **2**, showed inhibitory of AchE with IC_50_ values between 11.25 ± 0.04 and 57.78 ± 1.92 g/mL. Twelve other alkaloids: lycoramine (**6**), galanthaminone (**28**, Figure 2) 6α-deoxytazettine (**18**), norpluviine diacetate, 3-*O*-acetyl-1,2-dihydrogalanthamine (**29** and **30**, Figure 2), heamanthamine (**5**), undulatine diol (**31**, Figure 3), tazettine (**3**), acetylnatalensine (=acetylhaemanthamine) (**32**, Figure 2), undulatine (**33**, Figure 3) 3-*epi*-macronine (**21**) and crinan-3-one (**34**, Figure 2), were isolated from the same organic extract [33]. All these alkaloids were tested for AchE inhibition. The results of the bioassay showed that lycoramine, norpluvine diacetate and 6α-deoxytazettine contributed to potential acetylcholinesterase inhibition of the plant extract [33].

Lycorine (**1**), which is the main alkaloid, as well as dehydroanhydrolycorine (**20**) and 1-*O*-acetyllycorine (**35**, Figure 2) were identified in the leaves and bulbs of *Crinum amabile*, *Crinum erubescens*, *Crinum moorei*, *Amaryllis belladonna* and *Zephyranthes carinata*, which are all Amaryllidaceae species collected in different regions of Merida State-Venezuela [34]. In addition, buphanisine (**36**, Figure 3) in the *C. amabile* and *C. moorei* bulb extracts and undulatine (**33**) in the bulb extract of *A. belladonna* were found [34]. The extract of *C. amabile* leaves showed the strongest inhibition of AChE and BuChE followed by *C. erubescens* leaves [34]. 

Lycorine, galanthamine, tazettine (**1**–**3**), and chlidanthine (**37**, Figure 3) were identified in the leaf extract of *Pyrolirion albicans*, which grows in the coastal region of southern Peru [35]. From the bulb extract of the same plant, lycorine (**1**), galanthamine (**2**), vittatine (**4**), *N*-demethylgalanthamine (**38**, Figure 2), crinine, montanine and pancracine (**39**–**41**, Figure 3) and sternbergine and hippeastrine (**42** and **43**, Figure 2) were isolated and identified [35]. Montanine (**40**), which is the predominant alkaloid in the bulb extract, and its derivative pancracine (**41**) showed anti-inflammatory and immunomodulatory [36], antioxidant and antimicrobial [37] properties. They also showed significant anxiolytic, antidepressant, anticonvulsant [38] and antirheumatic activities [39]. 

*A. belladonna* has a geographical distribution covering mainly southern Africa [40], where it has significant usage in the traditional medicine of the native people but which was also isolated in Brazil [40]. The chemical analysis of the bulb organic extract of the samples collected in Brazil helped to identify twenty-six different AA, and three of them, namely, 1-*O*-acetylcaranine (**44**, Figure 2), buphanamine, and 3-*O*-acetylhamayne (**45** and **46**, Figure 3) were isolated [40]. The AA and the crude bulb organic extracts were tested against four different parasitic protozoa (*Trypanosoma cruzi*, *Trypanosoma brucei rhodesiense*, *Leishmania donovani*, and *Plasmodium falciparum* [40]). The crude organic extract and 3-*O*-acetylhamayne exhibited good antiprotozoal activity in vitro, although both had a high cytotoxic index [40]. 

Lycorine (**1**), 7-demethoxy-9-*O*-methylhostasine (**47**, Figure 3), and rutin were identified in the *Hippeastrum stapfianum* leaf extract [41]. This Amaryllidacea is an endemic species from the Brazilian savannah with biological and pharmacological activities, including AChE inhibition showing IC_50_ values ranging between 386.00 ± 0.97 and 114.80 ± 0.95 µg/mL [42]. Their potential for the treatment of Alzheimer’s disease is based on the ability of the plant extract to activate PPAR-α (peroxisome proliferator-activated receptor alpha) and PPAR-γ (peroxisome proliferator-activated receptor gamma) selectively [41].

Lycorine, galanthamine, lycoramine, galanthine, anhydrolycorine, didehydroanhydrolycorine and hippeastrine, (**1**, **2**, **6**, **7**, **17**, **20** and **43**,) norlycoramine, assoanine, and 9-*O*-demethylholycorine (**48** and **50**, Figure 2) and pancratinine C (**51**, Figure 3) were isolated from the bulb organic extract of *Ismene amancaes*, an endemic Peruvian Amaryllidaceae, which was collected during the flowering period in the surroundings of the town Pagash Alto, Salpo District, Otuzco Province, La Libertad Region (Peru) [43]. The crude plant extract also showed low inhibition of the enzymes AChE and BuChE, with IC_50_ values of 14.6 ± 0.6 and 37.6 ± 1.4 µg/mL, respectively, and good to moderate inhibitory activity against *Plasmodium falciparum* strain FCR-3 (chloroquine-resistant), the protozoan responsible of malaria disease, with IC_50_ value of 3.78 ± 0.3 µg/mL [43].

## 3. Alkaloids Isolated from Amaryllidacae Plants Collected in South Africa

**Figure 4:**
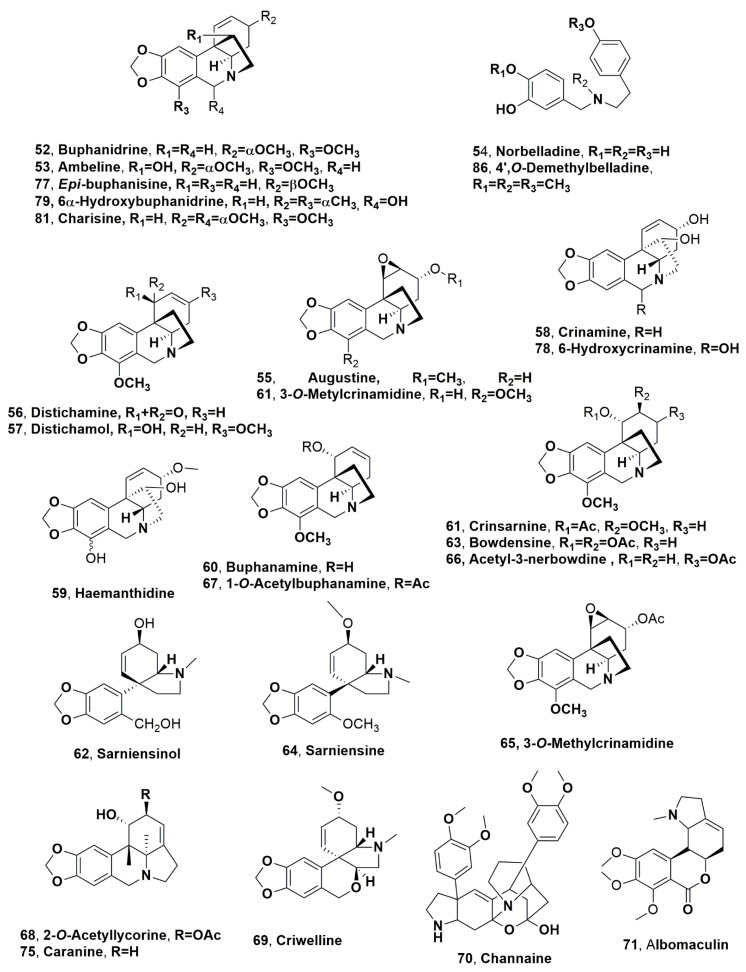
Alkaloids produced by South Africa Amaryllidaceae plants: *Boophone haemanthoides* (**52**–**60**), *Nerine sarniensis* (**61**–**64**), *Boophone disticha* (**65**–**67**), *Crinum buphanoides* (**68**), *Crinum graminicola* (**69**), *Sceletium tortuosum* (**70**), *Haemanthus humilis* (**71**), *Ammocharis coranica* (**75**, **77**–**79** and **81**), and *Clivia miniata* (**86**).

Lycorine, galanthamine, tazettine haemanthamine, homolycorine, ismine, trisphaeridine, undulatine, buphanisine, and crinine montanine (**1**–**3**, **5**, **10**, **12**, **13**, **33**, **36**, **39** and **40**) and buphanidrine, ambelline, norbelladine, augustine, disticahamine, distichaminol, crinamine, haemanthidine, and buphanamine (**52**–**60**, Figure 4) were isolated together with pancratistatine, an isocarbostyryl (see below Section 5), from *Boophone haemanthoides* [44]. The bulbs of this Amaryllidacea were collected during the flowering season in the Nieuwoudtville area of the Northern Cape Province of South Africa [44]. *B. haemanthoides* belong to the African genus *Boophone* Herb, which also includes *Boophone disticha*. *B. disticha* is widely distributed in Africa, ranging from Sudan to the Western Cape Province, while *B. haemanthoides* is a rare and endangered species found in a more limited range in the winter rainy region of South Africa, which is confined to a relatively small area in the southwest, the Western Cape area, where gentle rain falls from May to August, but the summers are dry, and in parts of southern Namibia [45]. Both Amaryllidaceae are widely used in folk medicine [46,47]. Among the alkaloids identified, lycorine (**1**) and distichamine (**56**) showed cytotoxic activity demonstrated in acute lymphoblastic leukemia (CEM), breast adenocarcinoma (MCF7) and cervical adenocarcinoma (HeLa) cells with IC_50_s in the range from 1.8 to 9.2 μM [44]. Previously from *B. heamanthoides*, collected in Saldhana Bay area in South Africa, also buphanisine crinine and buaphanidrine (**36**, **38** and **52**) were isolated together with distichamine (**56**) [48].

Crinsarnine and sarniensinol (**61** and **62**, Figure 4), belonging, respectively, to the crinine and mesembrine-types subgroups, were isolated together with bowdensine, sarniensine (**63** and **64**, Figure 4), and 1-*O*-acetyl-lycorine (**35**), from the dried bulbs of *Nerine sarniensis* [49], a species restricted to the Western Cape of the South Africa region [2]. All the alkaloids were assayed together with tazettine and 3-*epi*-macronine (**3** and **21**) against the Orlando reference strain of *Aedes aegypti*, which is the primary vector of dengue and yellow fever and Zika viruses [49]. The latter causes microcephaly and other serious brain anomalies during pregnancy, and it could easily become a potential threat to international public health safety [50]. Mosquito control is one of the main methods used to reduce the spread of these viruses. None of the compounds tested showed mortality against the first instar *Ae. aegypti* larvae at the concentrations tested. In adult topical bioassays, only crinsarnine (**61**) exhibited adulticidal activity with an LD_50_ value of 2.29 ± 0.049 µg/mosquito [49]. Regarding the structure–activity relationship, the pretazettine and crinine scaffold in alkaloids **62** and **64** and in **61** and **63**, respectively, proved to be important for their activity, while the pyrrole[de]phenanthridine scaffold present in alkaloid **35** appeared to not be important for toxicity [49]. Among the pretazettine group compounds, the opening of the B ring or the presence of a B ring lactone as well as the *trans*-stereochemistry of the A/B ring junction are important features for activity, while in crinine-type alkaloids, the substituent at C-2 seems to play a role in their toxicity [49].

Buphanidrine (**52**) is the main alkaloids isolated from the organic extract of *Boophone disticha* [51,52,53,54,55,56,57,58,59]. *B. disticha* is one of the most popular bulbous plants widely used in traditional medicine in South Africa [52]. Other known alkaloids isolated from the same organic extract were buphanisine, buphanamine, (**35** and **45**) 3-*O*-methylcrinamidine, and acetyl-3-nerbowdine (**65** and **66**, Figure 4) [60]. Later, 1-*O*-acetylbuphanamine (**67**, Figure 4) was also isolated from the same organic extract [61]

Haemanthamine and haemanthidine (**5** and **59**), together with metolachlor, which is an unusual chlorinated amide, were isolated from *Scadoxus puniceus* [62], an *Amaryllidaceae* species used in folk medicine as an herbal tonic prescribed to treat several ailments [62]. It is used as a detoxifying and energizing agent as well as to clear skin conditions, treat kidney and urinary infections, cure tonsillitis, and treat pneumonia in South Africa. Haemanthamine, haemanthidine, and metolachlor showed strong acetylcholinesterase inhibition with IC_50_ values of 23.1, 23.7, and 11.5 μM, respectively [62].

The alkaloids produced from *Crinum buphanoides*, *Crinum graminicola*, *Cyrtanthus mackenii*, and *Brunsvigia grandiflora*, which are all indigenous to South Africa, were isolated and identified [63]. Lycorine (**1**) appeared to be the main alkaloid produced by all four species, but *C. graminicola* was the highest alkaloid-producer plant (2 g/kg) [63]. Furthermore, *C. buphanoides* produced tazettine, 1-*O*-acetyllycorine (**3** and **35**), and 2-*O*-acetyllycorine (**68**, Figure 4), while haemanthamine, haemanthidine (**5** and **59**), and criwelline (**69**, Figure 4) were isolated from *C. graminicola*, and tazettine and 11-hydroxyvittatine (**3** and **8**) were produced by *C. mackenii* [63]. The latter alkaloid (**8**) and crinamine (**58**) were produced by *B. grandiflora* [63].

Channaine (**70**, Figure 4), which is an AA with an unusual cage-like ring structure at the interface of two aryl-hydroindole subunits, was isolated from *Sceletium tortuosum* [64]. This species was collected from St. Helena in the Western Cape Province of South Africa and belongs to the *Sceletium* genus, which is endemic to South Africa and which is a well-known producer of alkaloids [65,66]. Alkaloid **70** was previously isolated from the same Amaryllidaceae [67], but only its empirical formula and some functional groups were assigned. Later, Popelak and Lettenbauer [68] described that channaine (**66**) contained two veratrole rings, being a dimer of two subunits and racemic of both channaine compounds.

**Figure 5:**
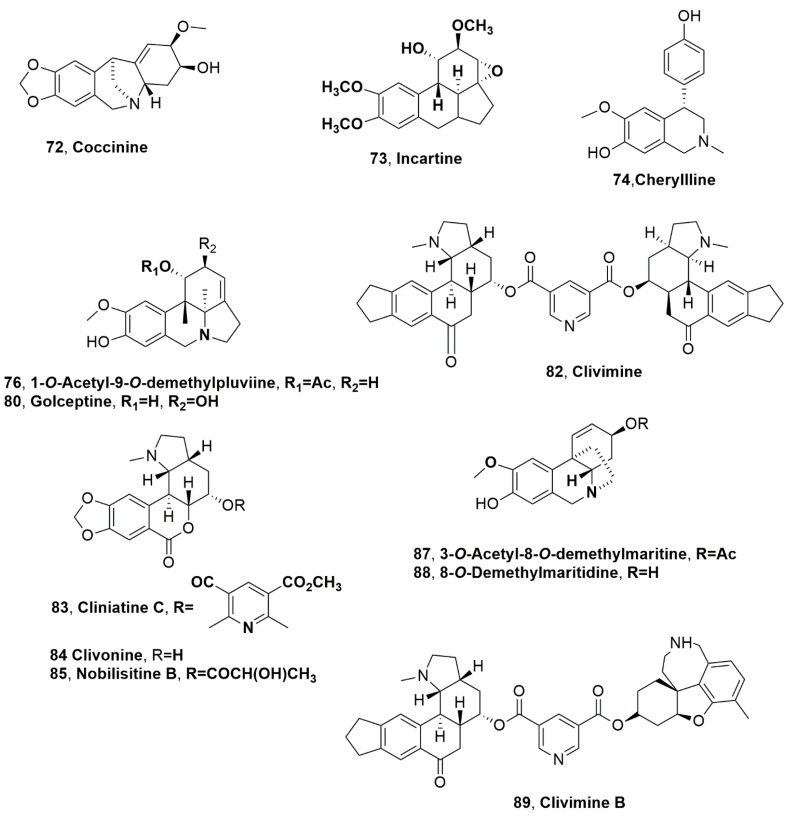
Alkaloids produced by South Africa Amaryllidaceae plants: *Haemanthus humilis* (**72** and **73**), *Crinum moorei* (**74**), *Ammocharis coranica* (**76**, **80** and **81**), and *Clivia miniata* (**82**–**85** and **87**–**89**).

Albomaculine (**71**, Figure 4), coccinine and incartine (**72** and **73**, Figure 5), and montanine (**40**) were isolated from *Haemanthus humilis*, which is indigenous to South Africa [69]. *H. humilis* does not synthesize lycorine, the main and most common alkaloid produced by this plant genus. Coccinine (**72**) appeared to be the main metabolite (1.49 g/kg). All alkaloids tested for their anticancer activity against a panel of six human cancer cell lines (the human breast MCF7 (HTB-22™), Hs578T (HTB-126™), and MDA-MB-231 (ATCC^®^HTB-26™), colon HCT-15 (CCL-225™), and lung A549 (CCL-185™) cancer cell lines as well as SK-MEL-28 (HTB-72™) melanoma cells, and coccinine and montanine (**72** and **40**) showed significant activity at low micromolar concentrations [69].

The ‘lily borer’ moth *Brithys crini* is a very dangerous parasite of Amaryllidaceae plants, having a great effect during the larval stage. The organic extract of *Crinum moorei*, collected in the botanical garden of the University of KwaZulu-Natal, was analyzed, and alkaloids belonging to different subgroups of AA were identified as ambelline (**53**) and cherylline (**74**, Figure 5). The presence of ambelline represents a surprise, as it was not previously isolated in the organic extract obtained from this Amaryllidaceae [70].

In the organic extract of *Ammocharis coranica* were identified lycorine, 1-*O*-acetylcaranine, crinamine (**1**, **44**, **58**) and caranine (**75**, Figure 4) [71]. Later, hamayne and 1-*O*-acetyllycorine (**11** and **35**) and 1-*O*-acetyl-9-*O*-demethylpluviine (**76**, Figure 5) were also isolated [72], as well as buphanisine, buphanidrine and ambelline (**36**, **52** and **53**), and *epi*-buphanisine and 6α-hydroxycrinamine (**77** and **78**, Figure 4) [73]. *A. coranica* (Ker Gawl.) Herb. is the second most widely distributed Amaryllidaceae of the Ammocharis Herb. genus and is found within all southern African countries, as in Little Karoo of South Africa, northward to Zimbabwe, and the southern Angola regions [74]. More recently, 6α-hydroxybuphanidrine and golceptine (**79**, Figure 4 and **80**, Figure 5) were isolated together with an unusual crinine type alkaloid such as charisine (**81**, Figure 4), from *A. coronica* [75].

Nineteen AA belonging to different subgroups were isolated from *Clivia miniata*, which is an herbaceous evergreen plant endemic to South Africa and Swaziland [76]. The alkaloids were identified as lycorine, galanthamine, tazettine, vittatine, haemanthamine 11-hydroxyvittatine, sternbergine, hippeastrine, 1-*O*-acetylcaranine, haemanthidine, caranine, (**1**–**5**, **8**, **42**–**44**, **59** and **75**), clivimine, cliniatine C, clivonine, nobilisitine B, 4′^,^O-demethylbelladine, 3-*O*-acetyl-8-*O*-demethylmaritidine, 8-*O*-demehylmaritidine, and clivimine B (**82**–**85**, Figure 5, **86**, Figure 4, and **89**, Figure 4) [76]. The main alkaloids were lycorine, haemanthamine, and clivimine (**1**, **5** and **82**). *C. miniata* is the most used Amaryllidacea species in folk medicine in South Africa [76]. All the isolated alkaloids were tested for their AChE/BuChE inhibition, using galanthamine and eserine as reference compounds. Alkaloids belong to the homolycorine structure type as clivimine, cliniatine C, clivonine, nobilisitine B and clivimine B, which show low AChE/BUChE inhibitory activity. Among the lycorine-type alkaloids such as lycorine, sternbergine, 1-*O*-acetylcaranine, and caranine was noted that the activity could be due to the presence of free hydroxyl groups in positions C1 and C2, which are not present in those of the homolycorine type. This diol system is probably a functional group that improves binding in the active site of AChE/BuChE. However, lycorine and several of its analogues did not show significant activity against AChE and BuChE [76].

## 4. Alkaloids Isolated from Amaryllidacae Plants Collected in Mediterranean Basin

**Figure 6:**
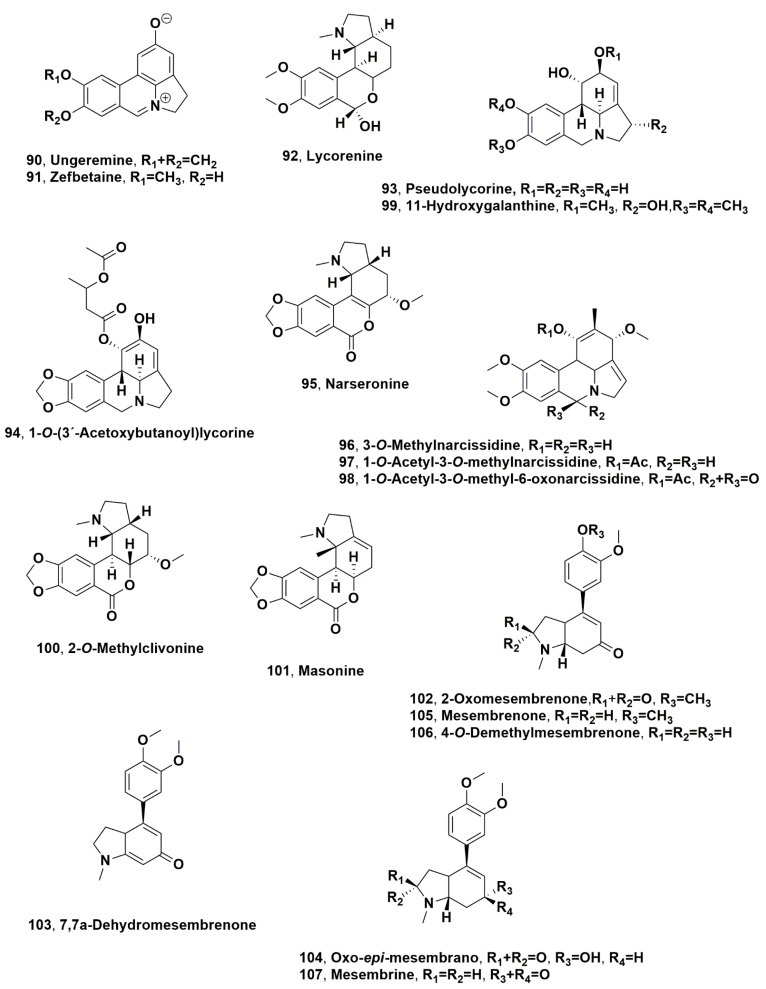
Alkaloids produced by Mediterranean Amaryllidaceae plants: *Pancratium maritimum* (**90**–**93**), *Narcissus serotinus* L. (**94**–**101**), and *Narcissus triandrus* (**102**–**107**).

Ungeremine and zefbetaine (**90** and **91**, Figure 6), which are two 2-oxyphenanthridinium alkaloids, were isolated from the bulb organic extract of Egyptian *Pancratium maritimum* collected from sandy hills on the northern coast during the flowering and fruit-producing stages [77]. From the same organic extract, lycorine, galanthamine, tazettine, haemanthamine, 11-hydroxyvittatine, homolycorine, trispheridine, pancracine, 9-*O*-demethylhomolycorine and haemanthidine, (**1**–**3**, **5**, **8**, **10**, **13**, **41**, **50** and **59**), and lycorenine (**92**, Figure 6) were isolated and identified [78,79]. Ungeremine showed toxicity against *Flavobacterium columnare* and *Edwardsiella ictaluri*, respectively, with IC_50_ and MIC values of 58 ± 0 and 3 ± 0 and (0.8 ± 0 and 0.9 ± 0.2 mg/L [80]. This activity was compared to that of lycorine (**1**) and pseudolycorine (**93**, Figure 6), isolated from *Narcisuss tazetta* subsp. *tazetta* [81], ungeremine isomer, zefbetaine (**90**), and anhdrolycorine (**17**). The aromatization of the C ring and the oxidation to an azomethine group of C-7 of the B ring are structural features important for antibacterial activity [82]. Furthermore, the presence of the 1,3-dioxole ring joined to the A ring and oxygen location of the C ring of the pyrrolo[de]phenanthridine skeleton also play significant roles on the antibacterial activity [82]. Ungeremine (**90**) showed to be a promising biofungicide against *Penicillium roqueforti* and *Aspergillus niger* with MIC90 values of 0.003 and 0.2 mg/mL [83]. These two fungi are very dangerous food contaminants and can cause bakery product deterioration. They show significant potential to be included as an appropriate biofilm that can be used in intelligent food packaging [83]. Alkaloid **90** was incorporated in chitosan-based microbeads, which were prepared by external gelation by using sodium tripolyphosphate (TPP) as a crosslinking agent. All the microbeads evidenced antimicrobial activity against *P. roqueforti* [84]. These microbeads were included in a thermoplastic starch-based polymer Mater-Bi (MBi), and MBi/CTUn and bioactive biocomposites were obtained. The films showed bioactivity against *P. roqueforti* [85].

1-*O*-(3’-acetoxybutanoyl)lycorine and narseronine (**94** and **95**, Figure 6) were isolated from *Narcissus serotinus*, an autumn flowering Amaryllidaceae collected in Ben Slimane, near Casablanca (Morocco) [86]. Successively from the same Amaryllidaceae but collected near Vinarós, Castellón Province (Spain), 3-*O*-methylnarcissidine and 1-*O*-acetyl-3-*O*-methylnarcissidine, 1-*O*-Acetyl-3-*O*-methyl-6-oxonarcissidine, 11-hydroxygalanthine and 2-*O*-methylclivonine (**96**–**100**, Figure 6), and the isocarbostyryl 2-methoxypratosine (see below Section 5) were isolated together, with incartine (**73**) masonine (**101**, Figure 6), galanthine and hippeastrine (**7** and **42**) [87].

**Figure 7:**
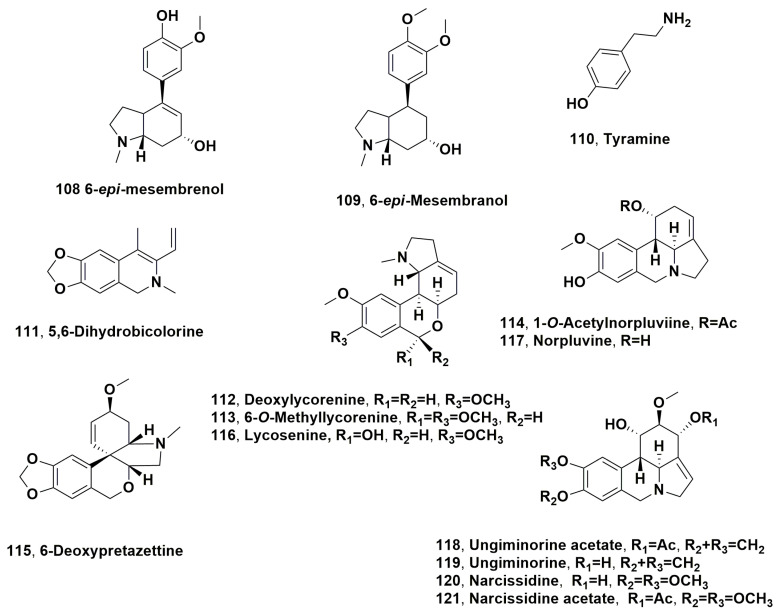
Alkaloids produced by Mediterranean Amaryllidaceae plants: *Narcissus triandrus* (**108** and **109**) and *Lapiedra martinezii* (**110**–**121**).

2-Oxomesembrenone, 7,7a-dehydromesembrenone and 2-oxo-epi-mesembrano (**102**–**104**, Figure 6) together with mesembrenone (**105**, Figure 6), which is the main alkaloid, 4-*O*-demethylmesembrenone and mesembrine (**106** and **107**, Figure 6), and 6-*epi*-mesembrenol and 6-*epi*-mesembranol (**108** and **109**, Figure 7) were isolated during the flowering period from *Narcissus triandus*, a wild daffodil of the Ganymedes section from the Iberian Peninsula [88]. The isolated alkaloid profile confirms the presence of mesembrane alkaloids in all the *Narcissus* taxa, as well as the absence of trace of typical alkaloids. Furthermore, the founding of mesembrane alkaloids in *Narcissus pallidulus* and some hybrids [89] showed an important phylogenetic aspect of *Narcissus*: the biosynthetic pathway of alkaloids by species belonging to the Ganymedes section is atypical among other Amaryllidaceae. In addition, mesembrane alkaloids produced by this group of Mediterranean daffodils share chemical features with the very distantly related dicotyledonous plants of the genus *Sceletium* from South Africa, whose potential therapeutic applications have already been recognized [88].

*Lapiedra martinezii* is an Amaryllidaceae species diffused in the Mediterranean basin, including essentially the Iberian Peninsula and the north of Africa [90], together with *Narcissus*, *Hannonia*, and *Vagaria gebìnera* [90,91]. *Lapiedra* is the oldest genus and is only diffused in the Mediterranean coastal side of the Baetic mountain chain (from Malaga to Valencia), with a few populations in the nearby Spanish city of Melilla (North Africa). The ability of *L. martinezii* to produce alkaloids is strictly related to its distribution area. Several alkaloids belonging to different subgroups were identified: as lycorine, tazettine, 11-hydroxyvittatine, homolycorine, ismine, trisphaeridine, kirkine, anhydrolycorine, didehydroanhydrolycorine, sternbergine, hippeastrine, assoanine, demethylhomolycorine, and lycorenine (**1**, **3**, **8**, **10**, **12**, **13**, **15**, **17**, **20**, **42**, **43**, **48**, **50** and **92**), as well as tyramine, 5,6-dihydrobicolorine, deoxylycorenine 6-*O*-methyllycorenine, 1-*O*-acetylnorpluviine 6-deoxypretazettine, lycosenine, norpluviine, ungiminorine acetate, ungiminorine, narcissidine, and narcissidine acetate (**110**–**121**, Figure 7) [92].

**Figure 8:**
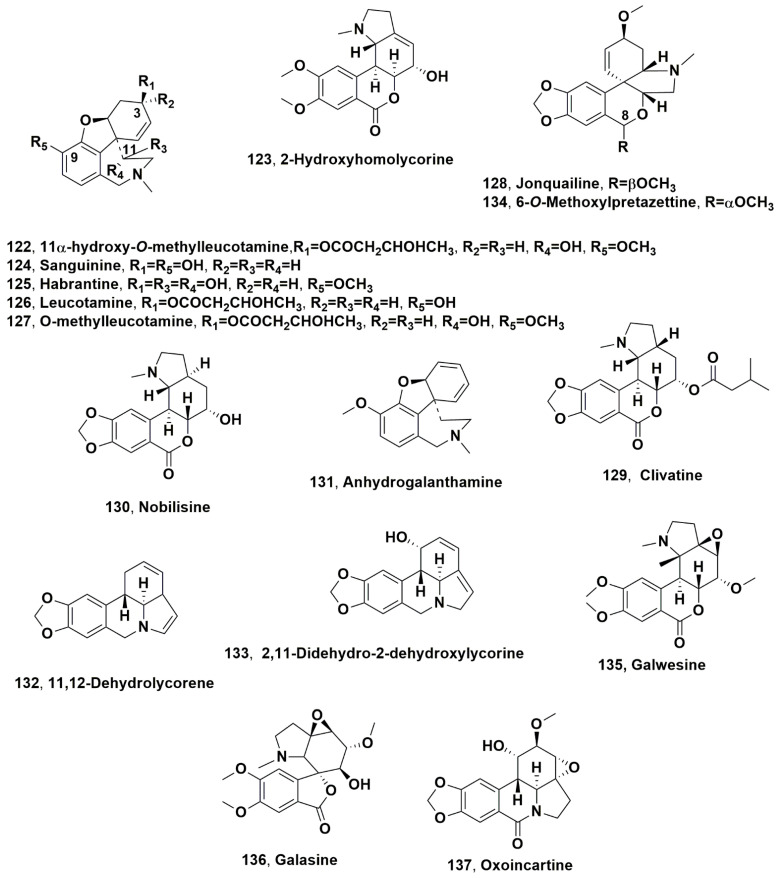
Alkaloids produced by Mediterranean Amaryllidaceae plants: *Pancratium illyricum* L. (**122**–**127**), *Narcissus jonquilla* quail (**128**), *Clivia nobilis* (**129** and **130**), and *Galanthus elwesii* (**131**–**138**).

11α-Hydroxy-*O*-methylleucotamine (**122**, Figure 8) was isolated together with 2-hydroxyhomolycorine, sanguinine, habranthine, leucotamine, and O-methylleucotamine (**123**–**127**, Figure 8) as well as lycorine, galanthamine, and vittatine (**1**, **2**, **4**), from fresh bulb extracts of *Pancratium illyricum* L. [93], a species endemic to Sardinia (Italy), which was collected during the flowering period in the South of the island of Punta San Michele, Cagliari, Italy [93]. Considering that the galanthamine type inhibits AChE [11], all the isolated alkaloids of the galanthamine type (**1**, **123**–**127**) showed good inhibitory activity against AChE, comparable to that of the positive control galanthamine hydrobromide [93]. The strongest inhibition was exhibited from 11α-hydroxy-*O*-methylleucotamine (**122**) and galanthamine hydrobromide, with IC_50_ values of 3.5 ± 1.1 and 1.5 ± 0.2 μM, respectively [93]. Alkaloids **123**-**127** showed a marked decrease in enzyme inhibition, while sanguine (**124**) showed the strongest activity (ca. 10 times higher than galanthamine [93]). Alkaloid **124**, compared with galanthamine, has a hydroxyl group at C-9 instead of a methoxyl group, which seems to be important for the inhibition of AChE contributing to its effective binding to the enzyme [94]. In addition, the spatial orientation of the hydrophilic group can affect this binding interaction. The β-configuration of the hydroxyl group at C-11 of 11β-hydroxygalanthamine decreases, by 10 times, the activity compared to its α-epimer habranthine (**125**) [95]. The low decrease in 11α-hydroxy-*O*-methylleucotamine (**122**), which showed an IC_50_ value of 1.61 ± 0.2, is probably due to the presence of the bulky butyryl group at C-3 [93].

Jonquailine (**128**, Figure 8), an alkaloid belonging to the pretazettine group, was isolated from dried bulbs of *Narcissus jonquilla* quail, widespread in Spain and Portugal [96]. Alkaloid **128** showed significant antiproliferative effects against glioblastoma, melanoma, uterine sarcoma and non-small-cell lung cancer cells, which exhibit various forms of drug resistance, including resistance to apoptosis and multi-drug resistance [97]. Jonquailine (**128**) was able to synergize, with paclitaxel, its antiproliferative action against drug-resistant lung cancer cells [97]. The hydroxylation at C-8 is an important feature for anticancer activity, but this seems to be affected by both the stereochemistry and acetalization of lactol [97].

Lycorine and 8-*O*-demethylmaritidine (**1** and **89**) were isolated together with clivatine and nobilisine (**129** and **130**, Figure 8) from the flower extract of *Clivia nobilis*, cultivated in Egypt [98]. The crude flower organic extract as well as all the alkaloids isolated were tested for their antibiotic activity against Gram-positive Staphylococcus aureus and Gram-negative *Pseudomonas aeroginosea* bacteria. The crude flower extract showed antibacterial activity against both microorganisms, while nobilisine (**130**) showed very good activity against the Gram-negative *P. aeroginosea* [98].

**Figure 9:**
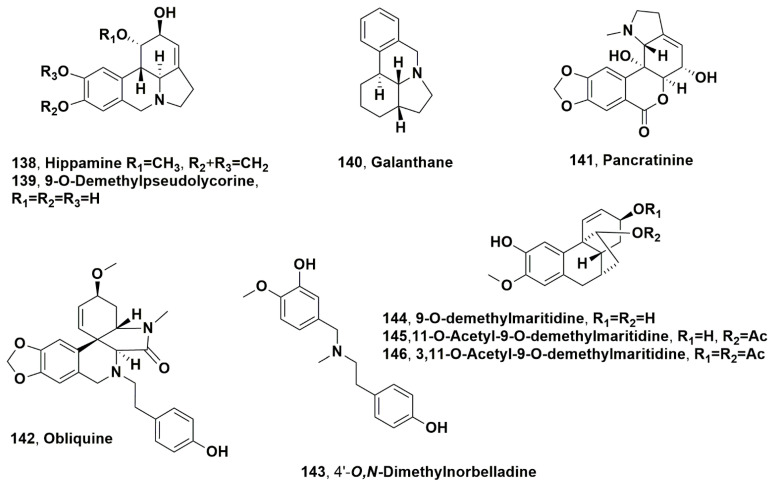
Alkaloids produced by Mediterranean Amaryllidaceae plants: *Galanthus elwesii* (**138**), *Narsissus tazetta a* subsp. *tazetta* (**139**), *Pancratium maritimum* (**140**–**142**), and *Galanthus fosteri* (**143**–**146**).

Lycorine, galanthamine, tazettine, haemanthamine, galanthine, 11-hydroxyvittatine, ismine, trispheridine, narwedine, galanthindole, anhydrolycorine 3-*epi*-macronime, crinan-3-one, crinine, hippeastrine, assoanine, 9-*O*-demethylhomolycorine, incartine, 8-*O*-demethylmaritidine, 5,6-dihydrobicolorine, saguinine, and O-methyleucotamine (**1**, **2**, **3**, **5**, **7**, **8**, **12**, **13**, **14, 16**, **17**, **21**, **34, 38**, **43, 48**, **50**, **73**, **88**, **111**, **124** and **127**) and anhydrogalanthamine, 11,12-dehydrolycorene, 2,11-didehydro-2-dehydroxylycorine, 6-*O*-methoxylpretazettine, galwesine, galasine, oxoincartine (**131**–**138**, Figure 8), and hippamine (**139**, Figure 9) were isolated from the aerial parts of *Galanthus elwesii*, collected from three different regions of Turkey [99].

Hippamine (**139**,) had been also previously extracted as a minor alkaloid from Sternbergia lutea [100]. The organic extract of samples of *G. elwessii* collected in Karaburun-Izmir showed to be the most active. A molecular docking study was carried out to determine the binding of alkaloids in the gorge of the active site of acetylcholinesterase (AChE) of *Electrophorus electricus*, and equine butyrylcholinesterase (BuChE) and O-methylleucotamine (**127**) showed the most interesting results [99].

Galanthamine (**2**) was produced by *Narcissus poeticus* collected in Abruzzo (Italy). Alkaloid **2** was found in all organs of the plant (flower, stem, bulb and root), but it accumulates in the bulbs [101]. The organic extract of *N. poeticus* obtained from the flowers is also used as an important fragrance in perfumery, while that of the petals and coronas show allergenic properties [101].

Lycorine (**1**) was isolated from the bulb extract of *Pancratium foetidum*, collected in Saïdia, Oujda region, Morocco [102]. Lycorine showed moderate antibacterial activity and had more efficacy than streptomycin and ampicillin against *P. aeruginosa*. A virtual docking ligand-lycorine protein screening showed that compound **1** can interact with target amino residues studied by hydrogen and metal-ion bonds [102].

Galanthamine, lycorine pseudolycorine and 11-hydroxygalanthine (**1**, **2**, **93** and **99**) were isolated from the bulb organic extract of *Narsissus tazetta* subsp. *tazetta*, collected during the flowering period in Akçapınar/Muğla, Turkey [81]. Other alkaloids were isolated and identified as narwedine, anhydrolycorine, 9-*O*-demethylhomolycorine, 4′,O-methylnorbelladine, 8-*O*-demethylmaritidine, pseudolycorine, 1-*O*-acetyl-3-*O*-methylnarcissidine, 11-hydroxygalanthine, narcissidine, saguinine (**14**, **17**, **50**, **86**, **88**, **93**, **97**, **99**, **120**, **124**) and 9-*O*-methylpseudolycorine (**139**, Figure 9) and pancratinine C (**51**) [81]. All the alkaloids isolated were assayed for their ability to inhibit AChE and BuAchE, and 11-hydroxygalanthine and narcissidine (**99** and **120**) showed significant activity on acetylcholinesterase (AChE) [81].

From the bulbs of *Pancratium maritimum*, which were always collected in Turkey, Pamucak/Aydın), several alkaloids such as lycorine, galanthamine, tazettine, 11-hydroxyvittatine, homolycorine, ismine, trispheridine, galantindole, 6-*O*-methyoxypretazzettine, dehydroanhydrolycorine, buphanisine, N-demethylgalanthamine crinine, pancracine, hippeastrine, assoanine, 8-*O*-demethylmaritidine, 9-*O*-demethylhomolycorine 5,6-dihydrobicolorine, and 2,11-didehydro-2-dehydroxylycorine (**1**, **2**, **3**, **8**, **10**, **12**, **13**, **16**, **19**, **20**, **36**, **38**, **39 41**, **43**, **48**, **88**, **90**, **111** and **133**) and galanthane, pancratinine, and obliquine (**140**, **141** and **142**, Figure 9) were isolated [103]. The crude bulb organic extract showed AChE and BuChE inhibition activity with IC_50_ values of 3.49 and 28.96 µg/mL, respectively [103]. From the same Amaryllidaceae, but collected in Squillace, Calabria region, Italy, ten Amaryllidaceae alkaloids were isolated and identified as tazettine, vittatine, haemanthamine, 11-hydroxyvittatine, homolycorine, pancracine, haemanthidine, and 9-*O*-demethyllycorine (**3**, **4**, **5**, **8, 10**, **41**, **59** and **90**), and obliquine (**141**) [104]. Haemanthidine (**59**) was isolated as a scalar mixture of two 6-epimers, as already known for other 6-hydroxycrinine alkaloids. Lycorine and haemanthidine (**1** and **59**) showed cytotoxic activity on Hacat cells and A431 and AGS cancer cells, while pancracine (**41**) exhibited selective cytotoxicity against A431 cells [104]. Alkaloids **2**, **5**, **41** and **59** also showed antiretroviral activity, inhibiting pseudotyped human immunodeficiency virus (HIV)−1 with EC_50_ values of 25.3 and 18.5 µM, respectively [104]. In addition, all the alkaloids were able to avoid dengue virus (DENV) replication with EC_50_ values ranging from 0.34 to73.59 µM at low non-cytotoxic concentrations (CC_50_ ranged from 6.25 µM to >100 µM [104]). Haemanthamine, pancracine, and haemanthidine (**5**, **41** and **59**) appeared to be the most potent anti-DENV inhibitors with EC_50_ values of 337, 357 and 476 nM, respectively [104].

Several alkaloids were isolated from *Galanthus fosteri*, diffused mainly in south- and north-central Turkey [105], such as lycorine, galanthamine, galanthine, 11-hydroxyvittatine, ismine, trispheridine, anhydrolycorine, didehydroanhydrolycorine crinine, assoanine, incartine, 5,6-dihydrobicolorine, galwesine, and oxoincartine 9-*O*-demethylpseudolycorine, (**1**, **2**, **7**, **8**, **12**, **13**, **17**, **20**, **38, 48**, **73**, **111**, **136**, and **139**), and O,N-dimethylnorbelladine, 9-*O*-demethylmaritidine, 11-*O*-acetyl-9-demethylmaritidine, and 3,11-*O*-diacetyl-9-*O*-demethylmarititidine (**143**, **144**, **145** and **146**, Figure 9) [106].

*Leucojum aestivum*, commonly named summer snowflake, is a bulbous plant in the Euro-Mediterranean region and is a well-known source of pharmacologically important alkaloids. Among all the alkaloids produced, galanthamine (**2**) is the major bioactive compound, as well as lycorine (**1**). *L. aestivum* is a salt-tolerant plant, and treatment with 4 g/L of CaCl_2_ increased the amount of galanthamine and the antioxidant activities [107]. Bulbs collected in six different locations in Turkey (Gölcük-Bolu, Yeniçağa-Bolu, Kaynarca-Sakarya, Delmece-Yalova, Uluabat-Bursa and Terkos-Istanbul) at three different growing periods showed that genetic factor is important in alkaloid biosynthesis in the bulbs collected in Gölcük-Bolu, which showed to be the most productive in both alkaloids **1** and **2**. The same alkaloids were obtained in abundant amounts from both the bulbs and leaves collected in Delmece-Yalova. The vegetative period followed by ripening are the two periods in which the amount of both alkaloids is high [108].

## 5. Isocarbostyryls Close to Some Amaryllidaceae Alkaloids

**Figure 10:**
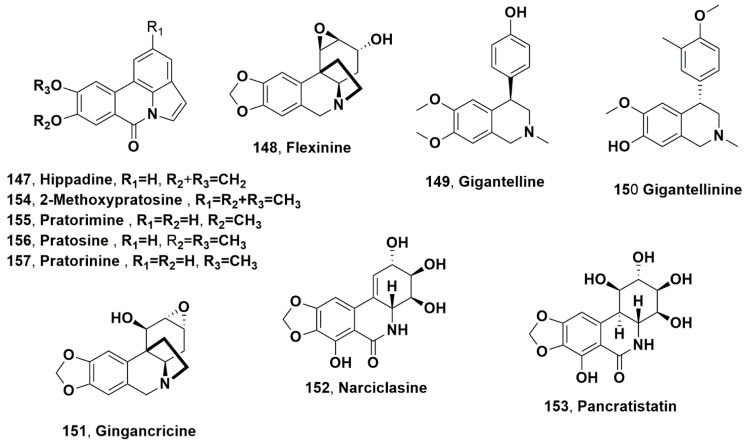
Isocarbostyryl isolated from *Pancratium maritimum*, *Nerine sarniensis*, *Crinum jagus*, and *Crinum buphanoides* (**147**), *Narcissus pseudonarcissus* and *Sternbergia lutea* (**152**), *Hymenocallis littoralis* and *Boophane haemanthoides* (**153**), *Narcissus serotinus* (**154**), *Crinum buphanoide* (**155**–**157**), and *Crinum latifolium* and *Crinum pretense* (**147** and **155**–**157**). Alkaloids isolated from *C. jagus* (**148**–**151**).

Hippadine (**147**, Figure 10) is an isocarbostyryl analogue of lycorine isolated together with the alkaloids ungeremine and zefbetaine (**89** and **90**) from Egyptian *P. maritimum*, as described in Section 4 [77]. Isocarbostyryl **147** was isolated together with 1-*O*-acetyl-lycorine, *crinsarnine, sarniensinol, bowdensine, and sarniensine (**35**, **61**–**64**) from bulbs of N. sarniensis*, as described in Section 2 [49]. In this work, compound **147** and the AA were tested for their mosquitocidal activity against Ae egypti. In adult mosquitos, it showed 23% of mortality [49]. Compound **147** was isolated together with lycorine, crinine, cherylline, and sanguinine (**1**, **38**, **74** and **124**), and flexinine, gigantelline, gigantellinine and gigancrinine (**148**–**151**, Figure 10) from the bulbs of *Crinum jagus* (syn. = *Crinum giganteum*) collected in Saint Louis, Senegal [109]. Cherylline, gigantellinine, crinine, flexinine and sanguinine showed inhibitor activity against AChE in a dose-dependent manner, and sanguinine had strong efficacy with an IC_50_ value of 1.83 ± 0.01 μM, while cherylline and hippadine showed weak cytotoxicity at 100 μM [109].

Among isocarbostyryls, narciclasine and pancratistatin (**152** and **153**, Figure 10) are amide analogues of lycorine that are very well known for they anticancer activity [6,110,111,112]. Narciclasine (**152**) was isolated together with haemamthamine (**5**) from the bulbs of *Narcissus pseudonarcissus* [113]. Previously, isocarbostyryl **152** was also isolated from *Sternbergia lutea*, its new process of extraction and purification was optimized, and its NMR spectroscopic full data were assigned [114]. Pancratistatin was extracted for the first time from *Hymenocallis littoralis* bulbs [115], and it was also isolated from *B. haemanthoides* bulbs, as reported above in Section 2 [1].

2-Methoxypratosine (**154**, Figure 10) was isolated, together with AA galanthine, hipeastrine, incartine, narseronine, 3-*O*-methylnarcissidine, 1-*O*-acetyl-3-*O*-methyl, masonine, and narcissidine (**7**, **43**, **73**, **95**, 97, **120**, and **101**) from the organic extract of fresh whole *N. serotinus*, as described in Section 4 [87].

Pratorimine (**155**, Figure 10) was isolated together with hippadine (**147**) and AA tazettine, 1-*O*-acetyl, and 2-*O*-acetyl-lycorine (**3**, **35** and **68**) from the bulb organic extract of *C. buphanoides* [63]. Isocarbostyryl **155** was previously isolated together with the two analogues pratosine and pratorinine (**156** and **157**, Figure 10) as well as hippadine and the AA lycorine and ambelline (**1** and **53**) from *Crinum latifolium* [116]. The isocarbostyryls **155** had been firstly isolated from *C. latifolium* and *Crinum pratense* Herb. [117].

## 6. Conclusions

This review describes the chemical and biological properties of alkaloids isolated in the last two decades from different Amaryllidaceae species. The different sections (Section 2, Section 3 and Section 4) report, in detail, the characterization of alkaloids isolated from Amaryllidaceae species diffused in regions of Southern Africa, Andean South America and the Mediterranean basin. The use of plant/alkaloids in folk medicine was also reported in the discussion, and in some cases, the relationships between structure and biological activity as well as their mode of action and biosynthetic pathway were also discussed. This review is divided into four sections, with the first three (Section 2, Section 3 and Section 4) chronologically reporting the alkaloids isolated in the three different cited areas. The results and corresponding studies are summarized in Table 1. The fourth section (Section 5) describes the chemical and the biological characterization of isocarbostyryl isolated from some *Amaryllidaceae* species in the last two decades in the same world regions, and the results and studies are summarized in Table 1. The content of review is an overview that could be very useful for scientists and readers interested in new pro-drug compounds with potential application in medicine.

## Figures and Tables

**Table 1:** Alkaloids and isocarbostyrls produced by *Amarylldaceae* spp. of Andean South America, South Africa, and the Mediterranean basin in the last two decades.

Alkaloid	Subgroup	Amaryllidaceae	Biological Activity	Reference
**AA from Andean South America**				
Lycorine (**1**)	Lycorane	*Habranthus jamesonii*, *Phycella herbertiana* and *Rhodophiala mendocina*	Not reported	[20]
			Cytotoxic, apoptotic, antiviral, antifungal, and antiprotozoan	[6,22]
			Anti-inflammatory	[24]
			Antileukemia	[25]
		*Caliphruria subedentata*	Not reported	[26,28]
		*Crinum amabile*, *Crinum erubescens*, *Crinum moorei*, *Amaryllis belladonna* and *Zephyranthes carinata*	“	[34]
		*Pyrolirion albicans*	“	[35]
		*Hippeastrum stapfianum*	“	[41]
		*Ismene amancaes*	“	[43]
		*Boophone haemanthoides*	Cytotoxic	[44]
		*Crinum buphanoides, Crinum graminicola, Cyrtanthus mackenii and Brunsvigia grandiflora*	Not reported	[63]
		*Ammocharis coranica*	“	[71]
		*Clivia miniata*	Inhibition of AChE/BUChE	[76]
		*Pancratium maritimum*	Not reported	[77,78]
		*Lapiedra martinezii*	Not reported	[92]
		*Pancratium illyricum*	“	[93]
		*Clivia nobilis*	“	[98]
		*Galanthus elwesii*	“	[99]
		*Pancratium foetidum*	Antibiotic	[102]
		*Narcissus tazetta* subsp. *tazetta*	Not reported	[81]
		*Galanthus fosteri*	“	[106]
		*Leucojum aestivum*	“	[107,108]
Galanthamine (**2**)	Galanthamine	*H. jamesonii*, *P. herbertiana*, *R. mendocina* and *Zephyranthes filifolia*	Inhibition of acetylcolinestarase	[20]
			Anticancer	[25]
		*C. subedentata*	Cytotoxic	[27,28]
		*Hippeastrum papilio*	Not reported	[32]
		*Rhodolirium andicola*	Inhibition of acetylcolinestarase	[33]
		*P. albicans*	Not reported	[35]
		*I. amancaes*	“	[43]
		*B. haemanthoides*	“	[44]
		*C. miniata*	Inhibion of AChE and BuChE	[76]
		*P. maritimum*	Not reported	[78,79]
		*Pancratium illyricum*	Inhibion of AChE and BuChE	[93]
		*G. elwesii*	“	[99]
		*Narcissus poeticus*	Not reported	[101]
		*N. tazetta* subsp. *tazetta*	Inhibion of AChE and BuChE	[81]
		*G. fosteri*	Not reported	[106]
		*Leucojum aestivum*	“	[107,108]
Tazettine (**3**)	Pretazettine	*H. jamesonii*, *P. herbertiana* and *R. mendocina*	Not reported	[20]
			Cytotoxic	[26]
		*C. subedentata*	Not reported	[27,28]
		*R. andicola*	“	[33]
		*P. albicans*	“	[35]
		*B. haemanthoides*	“	[44]
		*C. miniata*	“	[76]
		*P. maritimum*	“	[78,79]
		*L. martinezii*	“	[92]
		*Crinum graminicola*	“	[63]
		*Cyrtanthus mackenii*	“	
		*G. elwesii*	“	[99]
Vittatine (**4**)	α-Crinane	*H. jamesonii, P. herbertiana and R. mendocina*	Not reported	[20]
			Cytotoxic and antibacterial	[23]
		*P. albicans*	Not reported	[35]
		*C. miniata*	“	[76]
		*P. illyricum*	“	[93]
Heamanthamine (**5**)	“	*H. jamesonii, P. herbertiana and R. mendocina*	Not reported	[20]
			Induced apoptosis	[22]
			Antimalarial	[23]
		*C. subedentata*	Not reported	[27,28]
		*H. papilio*	“	[32]
		*B. haemanthoides*	“	[44]
		*R. andicola*	“	[33]
		*Scadoxus puniceus*	Inhibition of AChE and BuAchE	[62]
		*C. graminicola*	“	[63]
		*C. miniata*	“	[76]
		*P. maritimum*	“	[78,79]
		*G. elwesii*	“	[99]
Lycoramine (**6**)	Galanthamine	*H. jamesonii*, *P. herbertiana*, *R. mendocina*	Not reported	[20]
		*R. andicola*	Inhibition of ACheE and BuChE	[33]
		*I. amancaes*	Not reported	[43]
Galanthine (**7**)	Lycorane	*H. amesonii* and *P. herbertiana*	Not reported	[21]
			Analgesic and hypotensive	[23]
		*N. serotinus*	Not reported	[87]
		*G. elwesii*	“	[99]
		*G. fosteri*	“	[106]
11-Hydroxyvittatine (**8**)	α-Crinane	*H. amesonii* and *P. herbertiana*	Not reported	[21]
			Cytotoxicity and antibacterial	[6]
		*Cyrtanthus mackenii*	Not reported	[63]
		*C. miniata*	“	[76]
		*P. maritimum*	“	[78,79]
		*L. martinezii*	“	[92]
		*G. elwesii*	“	[99]
		*G. fosteri*	“	[106]
Maritidine (**9**)	α-Crinane	*C. subedentata*	Not reported	[27,28]
Homolycorine (**10**)	Homolycorine	“	“	“
		*B. haemanthoides*		[44]
		*L. martinezii*	“	[92]
		*G. elwesii*	“	[99]
		*G. fosteri*	“	[106]
Hamayne (**11**)	α-Crinane	*C. subedentata*	“	[31]
		*A. coranica*	“	[56]
Ismine (**12**)	Ismine	*C. subedentata*	“	[31]
		*B. haemanthoides*	“	[44]
		*L. martinezii*	“	[92]
		*G. elwesii*	“	[99]
		*G. fosteri*	“	[106]
Trisphaeridine (**13**)	Narciclasine	*C. subedentata*	“	[31]
		*B. haemanthoides*	“	[44]
		*L. martinezii*	“	[92]
		*G. elwesii*	“	[99]
		*G. fosteri*	“	[106]
Narwedine (**14**)	Galanthamine	*C. subedentata*	“	[31]
		*H. papilio*	“	[32]
		*G. elwesii*	“	[99]
Kirkine (**15**)	Lycorine	*C. subedentata*	“	[31]
		*L. martinezii*	“	[92]
Galanthindole (**16**)	Ismine	*C. subedentata*	“	[31]
		*G. elwesii*	“	[99]
		*G. fosteri*		[106]
Anhydrolycorine (**17**)	Lycorine	*I. amancaes*	“	[43]
		*L. martinezii*	“	[92]
		*G. elwesii*	“	[99]
		*N. tazetta* subsp. *tazetta*	“	[81]
		*G. fosteri*	“	[106]
6α-Deoxytazettine (**18**)	Tazettine	*C. subedentata*	“	[31]
		*R. andicola*	Inhibition of acetylcolinestarase	[33]
6-Methoxypretazettine (**19**)	“	*C. subedentata*	Not reported	[31]
Dehydroanhydrolycorine (**20**)	Lycorane	“	“	“
		*C. amabile*, *C. erubescens*, *C. moorei*, *A. belladonna* and *Z. carinata*	“	[34]
		*I. amancaes*	“	[43]
		*L. martinezii*	“	[92]
		*P. maritimum*	“	[99]
3-*epi*-Macronine (**21**)	Tazettine	*C. subedentata*	“	[31]
		*R. andicola*	“	[33]
		*Galanthus elwesii*		[99]
Hippapiline (**22**)	Homolycorine	*H. papilio*	“	[32]
Papiline (**23**)	“	“	“	“
3-*O*-Demethyl-3-*O*-(3-hydroxybutanoyl)- haemanthamine (**24**)	α-Crinane	“	“	“
11β-Hydroxygalanthamine (**25**)	Galanthamine	“	“	“
Apogalanthamine (**26**),	Miscellaneous	“	“	“
9-*O*-Demethyllycosinine B (**27**)	Ismine	“	“	“
Galanthaminon (**28**)	Galanthamine	*R. andicola*	Not reported	[33]
Norpluviine diacetate (**29**)	Lycorane	“	Inhibition of acetylcolinestarase Antibacterial	“
3-*O*-Acetyl-1,2-dihydrogalanthamine (**30**)	Galanthamine	“	Not reported	“
Undulatine diol (**31**)	β-Crinane	“	“	“
Acetylnatalensine (**32**)	α-Crinane	“	“	“
Undulatine (**33**)	β-Crinane	“	“	“
		*B. haemanthoides*	“	[44]
		*A. belladonna*		[34]
Crinan-3-one (**34**)	α-Crinane	*R. andicola*	“	[33]
		*G. elwesii*	“	[99]
1-*O*-Acetyllycorine (**35**)	Lycorine	*C. amabile*, *C. erubescens*, *C. moorei*, *A. belladonna* and *Z. carinata*	Not reported	[34]
		*N. sarniensis*	“	[43]
		*C. buphanoides*	“	[63]
Buphanisine (**36**)	β-Crinine	*C. amabile* and *C. moorei*	“	[34]
		*B. haemanthoides*	“	[44]
		*B. disticha*	“	[61]
Chlidanthine (**37**)	Miscellanea	*Pyrolirion albicans*	Not reported	[35]
Crinine (**38**)	β-Crinine	“	“	“
		*B. haemanthoides*	“	[44]
		*G. elwesii*	“	[99]
		*G. fosteri*	“	[106]
*N*-Demethyl galanthamine (**39**)	Galanthamine	*P. albicans*	Not reported	[35]
		*P. maritimum*	“	[99]
Montanine (**40**)	Montanine	*P. albicans*	“	[35]
			Anti-inflammatory and immunomodulatory	[36]
			Antioxidant and antimicrobial	[37]
			Anxiolytic, antidepressant, and anticonvulsant	[38]
			Antirheumatic	[39]
		*B. haemanthoides*	Not reported	[44]
		*Haemanthus humilis*	Anticancer	[69]
Pancracine (**41**)	“	*P. albicans*	No reported	[35]
			Anti-inflammatory and immunomodulatory	[36]
			Antioxidant and antimicrobial	[37]
			Anxiolytic, antidepressant, and anticonvulsant	[38]
			Antirheumatic	[39]
Sternbergine (**42**)	Lycorane	“	Not reported	[35]
		*L. martinezii*	“	[92]
Hippeastrine (**43**)	Homolycorine	*P. albicans*	“	[35]
		*L. martinezii*	“	[92]
		*Galanthus elwesii*	“	[99]
1-*O*-Acetylcaranine (**44**)	Lycorane	*A. belladonna*	“	[40]
		*A. coranica*	“	[72]
Buphanamine (**45**)	β-Crinine	*A. belladonna*	“	[40]
3-*O*-acetylhamayne (**46**)	α-Crinine	“	Antiprotozoal, cytotoxic	““
7-Demethoxy-9-*O*-methylhostasine (**47**)	“	*Hippeastrum stapfianum*	Inhibition of AChE	[41]
Assoanine (**48**)	Lycorine	*I. amancaes*	Not reported	[43]
Norlycoramine (**49**)	Galanthamine	“	“	“
9-*O*-Demethylhomolycorine (**50**)	Homolycorine	*P. maritimum*	“	[78,79]
		*L. martinezii*	“	[92]
		*G. elwesii*	“	[99]
		*N. tazetta* subsp. *tazetta*	“	[81]
Pancratinine C (**51**)	Montanine	*P. maritimum*	“	[99]
**AA from South Africa**				
Buphanidrine (**52**)	“	*B. haemanthoides*	“	[44]
		*B. disticha*	“	[61]
		*A. coronarica*	“	[73]
Ambelline (**53**)	“	*B. haemanthoides*	“	[44]
		*C. moorei*	“	[70]
		*C. latifolium*	“	[117]
Norbelladine (**54**)	Norbelladine	*B. haemanthoides*	“	[44]
Augustine (**55**)	“	“	“	“
Distichamine (**56**)	“	“	Cytotoxic	“
Distichaminol (**57**)	“	“	Not reported	“
Crinamine (**58**)	α-Crinine	“	“	“
		*B. grandiflora*	“	[63]
Haemanthidine (**59**)	“	*B. haemanthoides*	“	[44]
		*S. puniceus*	Inhibition of acetylcholinesterase	[62]
		*C. graminicola*	Not reported	[63]
Buphanamine (**60**)	β-Crinine	*A. belladona*	“	[40]
		*B. haemanthoides*	“	[44]
		*B. disticha*	“	[61]
Crinsarnine (**61**)	“	*N. sarniensis*	Insecticidal	[43]
Sarniensinol (**62**)	Mesembrine	“	Not toxic	“
Bowdensine (**63**)	β-Crinine	“	“	“
Sarniensine (**64**)	Mesembrine	“	“	“
3-*O*-methylcrinamidine (**65**)	β-Crinine	*B. disticha*	Not reported	[61]
Acetyl-3-nerbowdine (**66**)	“	“	“	“
1-*O*-acetylbuphanamine (**67**)	“	“	“	“
2-*O*-Acetyllycorine (**68**)	Lycorane	*C. buphanoides*	“	[63]
Criwelline (**69**)	Tazettine	*C. graminicola*	“	“
Channaine (**70**)	Mscellanea	*Sceletium tortuosum*	“	[64]
Albomaculine B (**71**)	Homolycorine	*H. humilis*	No activity	[69]
Coccinine (**72**)	Montanine	“	Anticancer	“
Incartine (**73**)	Lycorane	“	No activity	“
		*N. serotinum*	Not reported	[87]
		*G. elwesii*	“	[99]
		*G. fosteri*	“	[106]
Cherylline (**74**)	Cherylline	*C. moorei*	“	[70]
Caranine (**75**)	Lycorane	*A. coranica*	“	[71]
1-*O*-Acetyl-9-*O*-demethylpluviine (**76**)	“	“	“	[72]
*epi*-Buphanisine (**77**)	β-Crinine	“	“	[81]
6α-Hydroxycrinamine (**78**)	“	“	“	“
6α-Hydroxybuphanidrine (**79**)	“	“	“	[75]
Golceptine (**80**)	Lycorane	“	“	“
Charisine (**81**)	β-Crinine	“	“	“
Clivimine (**82**)	Clivimine	*C. miniata*	Not reported	[76]
Cliniatine C (**83**)	Homolycorine	“	Inhibition of AChE/BuChE	“
Clivonine (**84**)	“	“	“	“
Nobilisitine B (**85**)	“	“	“	“
4′,O-Demethylbelladine (**86**)	Norbelladine	“	Not reported	“
3-*O*-Acetyl-8-*O*-demethylmaritidine (**87**)	α-Crinine	“	“	“
8-*O*-Demethylmaritidine (**88**)	“	“	“	“
		*Clivia nobilis*	“	[98]
		*Galanthus elwesii*	“	[99]
Clivimine B (**89**)	Clivimine	*Clivia miniata*	Not reported	[76]
**AA fromMediterannean Basin**				
Ungeremine (**90**)	Lycorane	*P. maritimum*	Not reported	[71]
			Antibacterial	[80,82]
			Antifungal	[83]
Zefbetaine (**91**)	“	“	Not reported	[71]
Lycorenine (**92**)	“	*P. maritimum*	“	[78,79]
		*L. martinezii*	“	[92]
Pseudolycorine (**93**)	Lycorane	*Narcisuss tazetta* subsp. *tazetta*	“	[81]
1-*O*-(3´-acetoxybutanoyl)lycorine (**94**)	“	*Narcissus serotinus*	“	[86]
Narseronine (**95**)	Homolycorine	“	“	“
3-*O*-Methylnarcissidine (**96**)	Lycorane	“	“	[87]
1-*O*-Acetyl-3-*O*-methylnarcissidine (**97**)	“	“	“	“
1-*O*-Acetyl-3-*O*-methyl-6-oxonarcissidine (**98**)	“	“	“	“
11-Hydroxygalanthine (**99**)	“	“	“	“
		*N. tazetta* subps. *tazetta*	Inhibition of AChEand BuAchE	[81]
2-*O*-Methylclivonine (**100**)	Homolycorine	*N. serotinus*	Not reported	[87]
Masonine (**101**)	Homolycorine	“	“	“
2-Oxomesembrenone (**102**)	Mesembrane	*Narcissus triandrus*	“	[88]
7,7a-Dehydromesembrenone (**103**)	“	“	“	“
Oxoepimesembrano (**104**)	“	“	“	“
Mesembrenone (**105**)	“	“	“	“
4-*O*-Demethylmesembrenone (**106**)		“	“	“
Mesembrine (**107**)	“	“	“	“
6-*epi*-Mesembrenol (**108**)	“	“	“	“
6-*epi*-Mesembranol (**109**)	“	“	“	“
Tyramine (**110**)	Alkylamide	*L. martinezii*	“	[92]
5,6-Dihydrobicolorine (**111**)	Miscellanea	“	“	“
		*G. elwesii*		[99]
		*P. maritimum*	“	[103]
		*G. fosteri*	“	[106]
Deoxylycorenine (**112**)	Homolycorine	*L. martinezii*	Not reported	[92]
6-*O*-Methyllycorenine (**113**)	“		“	“
1-*O*-Acetylnorpluviine (**114**)	“	“	“	“
6-Deoxypretazettine (**115**)	“	“	“	“
Lycosenine (**116**)	Homolycorine	“	“	“
Norpluviine (**117**)	Lycorane	“	“	“
Ungiminorine acetate (**118**)		“	“	“
Ungiminorine (**119**)		“	“	“
Narcissidine (**120**)		“	“	“
Narcissidine acetate (**121**)		“	“	“
11α-hydroxy-*O*-methylleucotamine (**122**)	Galanthamine	*Pancratium illyricum*	Inhibition of acetylchlinestarase	[93]
2-Hydroxyhomolycorine (**123**)	Homolycorine	“	No activity	“
Sanguinine (**124**)	Galanthamine	“	Inhibition of acetylchlinestarase	“
		*G. elwesii*	Not reported	[99]
Habranthine (**125**)	“	*P. illyricum*	Inhibition of acetylchlinestarase	[93]
Leucotamine (**126**)	“	“	“	“
*O*-Methylleucotamine (**127**)	“	“	“	“
		*G. elwesii*	“	[99]
Jonquailine (**128**)	Tazettine	*Narcissus jonquilla quail*	Anticancer	[97]
Clivatine (**129**)	Homolycorine	*C. nobilis*	No activity	[98]
Nobilisine (**130**)	“	“	Antibiotic	“
Anhydrogalanthamine (**131**)	Galanthamine	*G. elwesii*	Not reported	[99]
11,12-Dehydrolycorene (**132**)	Lycorane	“	“	“
2,11-Didehydro-2-dehydroxylycorine (**133**)	“	“	“	“
6-*O*-Methoxylpretazettine (**134**)	Tazettine	“	“	“
Galwesine (**135**)	Lycorane	*G. elwesii*	“	[99]
		*G. fosteri*	“	[106]
Galasine (**136**)	Homolycorine	*G. elwesii*	“	[99]
Oxoincartine (**137**)	Lycorane	“	“	“
		*G. fosteri*	“	[106]
Hippamine (**138**)	Tazettine	G. wlwesi	“	[99]
		*S. lutea*	“	[100]
9-*O*-demethylpseudolycorine (**139**)	Lycorane	*P. maritimum*	“	[99]
Galanthane (**140**)	Miscellaneous	“	“	“
Pancratinine (**141**)	Homolycorine	“	“	“
Obliquine (**142**)	Tazettine	“	Antiviral	[104]
*O,N*-Dimethylnorbelladine (**143**)	Norbelladine	*G. fosteri*	Not reported	[106]
9-*O*-Demethylmaritidine (**144**)	α-Crinine	“	“	“
11-*O*-Acetyl-9-*O*-demethylmaritidine (**145**)	“	“	“	“
3,11-*O*-Diacetyl-9-*O*-demethylmarititidine (**146**)	“	“	“	“
**Isocarbostyryls close to some Amaryllidaceae alkaloids**				
**Isocarbostyryl**	**Soubgroup**	**Amar yllidaceae**	**Biological Activity**	**Reference**
Hippadine (**147**)	Lycorane	*P. maritimum*	Not reported	[71]
		*N. sarniensis*	Insecticidal	[43]
		*C. jagus*	Cytotoxic	[109]
Flexinine (**148**)	β-Crinine	*Crinum jagus*	Inhibition of AChE	[109]
Gigantelline (**149**)	Cherylline	“	“	“
Gigantellinine (**150**)	“	“	“	“
Gigancrinine (**151**)	β-Crinine	“	“	“
Narciclasine (**152**)	Lycorine	*N. peudonarcissus*	Anticancer	[6,110,111,112,113]
		*S. lutea*	“	[114]
Pncratistatin (**153**)	“	*Hymenocallis littoralis*	“	[110,115]
2-Methoxypratosine (**154**)	“	*N. serotinus*	“	[87]
Pratorimine (**155**)	“	*C. buphanoides*	Not reported	[63]
Pratosine (**156**)	“	*C. latifolium*	“	[116]
Pratorinine (**157**)	“	“	“	“

“ means the same content.

## Data Availability

All the data reported in this review were based on SciFinder research using appropriate key words.
